# Sympathetic nervous system responses during complex walking tasks and community ambulation post-stroke

**DOI:** 10.1038/s41598-023-47365-5

**Published:** 2023-11-16

**Authors:** Kanika Bansal, David J. Clark, Emily J. Fox, Dorian K. Rose

**Affiliations:** 1 Department of Physical Therapy, University of Mount Union, 1972, Clark Ave, Alliance, OH 44601-3993 USA; 2 University of Florida, Gainesville, FL USA; 3 Brain Rehabilitation Research Center, Malcolm Randall Veterans Affair Medical Center, Gainesville, FL USA; 4 Brooks Rehabilitation, Jacksonville, FL USA

**Keywords:** Neuroscience, Psychology, Health care, Health occupations, Neurology

## Abstract

Stroke survivors frequently report increased perceived challenge of walking (PCW) in complex environments, restricting their daily ambulation. PCW is conventionally measured through subjective questionnaires or, more recently, through objective quantification of sympathetic nervous system activity during walking tasks. However, how these measurements of PCW reflect daily walking activity post-stroke is unknown. We aimed to compare the subjective and objective assessments of PCW in predicting home and community ambulation. In 29 participants post-stroke, we measured PCW subjectively with the Activities-specific Balance Confidence (ABC) Scale and objectively through electrodermal activity, quantified by change in skin conductance levels (SCL) and skin conductance responses (SCR) between outdoor-complex and indoor-steady-state walking. High-PCW participants were categorized into high-change SCL (ΔSCL ≥ 1.7 μs), high-change SCR (ΔSCR ≥ 0.2 μs) and low ABC (ABC < 72%) groups, while low-PCW participants were categorized into low-change SCL (ΔSCL < 1.7 μs), low-change SCR (ΔSCR < 0.2 μs) and high-ABC (ABC ≥ 72%) groups. Number and location of daily steps were quantified with accelerometry and Global Positioning System devices. Compared to low-change SCL group, the high-change SCL group took fewer steps in home and community (*p* = *0.04*). Neither ABC nor SCR groups differed in home or community steps/day. Objective measurement of PCW via electrodermal sensing more accurately represents home and community ambulation compared to the subjective questionnaire.

## Introduction

Stroke is a major leading cause of disability world-wide, afflicting more than 795,000 people in the United States of America every year^1^. The southeastern region of USA, including north Florida^2^, is often referred to as ‘the stroke belt’ due its 20–32% higher prevalence of stroke than the national average^3^. Most individuals regain the ability to walk in safe and predictable environments, such as their home, within 6 months post-stroke^4^, however, as many as 74% report dissatisfaction with their level of community ambulation outside of their home environment^5^. Stroke survivors frequently perceive community ambulation as challenging^6–8^ and report reduced balance confidence in accomplishing common, yet complex tasks such as negotiating uneven terrains and walking in unpredictable, crowded environments^5,8^. Perceived challenge of walking is a broad term that encompasses fear of falling, balance confidence, and anxiety specifically pertaining to walking-related activities^6,9^. The prevalence of increased perceived challenge of walking varies between 32 and 83% between the first six months to over four years post-stroke^10,11^. In fact, increased perceived challenge of walking triggers a fear-avoidance cycle^12,13^, leading to diminished self-reported community ambulation^14^, total daily walking activity^8,15–17^ and community participation^13,18,19^, limitations beyond those simply secondary to post-stroke physical impairments^20^. Thus, addressing perceived challenge of walking is vital for improving community ambulation, an essential yet unmet goal for more than 90% stroke survivors^21,22^.

Conventionally, the construct of perceived challenge of walking has been measured through self-report questionnaires that assess one’s confidence to perform daily activities without falling. For example, the modified Falls-Efficacy questionnaire^23^ and the Activities-Specific Balance Confidence (ABC) Scale^24^ evaluate balance confidence during standing- and walking-related activities in and around one’s home/yard and community environment. While these standardized questionnaires are validated, easy to administer, inexpensive and time efficient, subjective measurement of perceived challenge comes with biases such as over-reporting positive traits, under-reporting negative traits or choosing extreme scores^25,26^. Moreover, since self-reported balance confidence is solely based on one’s own interpretation of their abilities to perform complex tasks, it does not always align with the physiological stresses of walking in challenging situations^6^. Since perceived challenge has a strong impact on community ambulation^5,13,16^, its assessment needs to be coupled with objective measures based on physiologic responses that may or may not be consciously perceived by an individual, such as quantification of sympathetic nervous system (SNS) activity^9,27–29^.

SNS is activated during physically or cognitively challenging and stressful situations, eliciting a ‘fight or flight’ response, causing an increase in eccrine sweat gland activity, elevated cardiopulmonary responses as well as a drop in skin temperature^27,30–32^. These increased physiological stress responses may be objectively quantified through continuous monitoring of pulse wave, skin temperature and skin conductance^31,32^. Arterial pulse wave monitoring quantifies an individual’s heart rate variability (HRV), which is known to decrease under stressful situations such as when walking in complex urban streets versus calming forest trails^31–34^. Skin temperature is also known to drop when viewing fearful graphics in young healthy adults^35^. However, both HRV measurement and skin temperature analysis have not yet been validated to measure autonomic stress responses during walking in individuals with stroke. Moreover, HRV and skin temperature are influenced by both sympathetic and parasympathetic nervous system whereas eccrine sweat gland activity in the palms and soles is primarily controlled by the SNS through sudomotor nerves and is less impacted by thermoregulatory changes^9,36^. In stressful situations, increased eccrine sweat gland activity leads to reduced resistance and increased electrical conductance of the skin, which can be easily measured with a small, imperceptible direct current via skin conductance^27,30^. Additionally, through a series of investigations Clark et al., and Chatterjee et al., have demonstrated the feasibility, utility, and validity of skin conductance measurement in quantifying SNS activity during challenging walking tasks in varied indoor and outdoor environments for both older adults^9,28^ and people post-stroke^6,37^.

Compared to indoor walking, stroke survivors demonstrated increased SNS responses during challenging outdoor tasks like walking up and down a ramp, negotiating a curb, and walking on grassy terrain^6,9,37^. Higher SNS activity during complex walking tasks is also associated with worse task performance, as quantified by slower walking speeds^6^. Thus, SNS activity reflects both the ‘conscious’ and ‘unconscious’ aspects of perceived challenge, fear of falling, as well as anticipation of possible negative consequences (e.g., falls), present when performing challenging tasks^9,28,29^. Moreover, SNS activity depends on several factors such as the demands of and an individual’s physical skill for completing the task, their past experience with and their perceived confidence in accomplishing the task successfully as well as the environment in which the task is performed^6^. These factors form important components of successful community ambulation, and may influence one’s decision to engage in community ambulation, outside the familiarity of their home^38^. Thus, unlike self-reported balance confidence^27^, assessing SNS responses via skin conductance allows for valid, objective, non-invasive, nonverbal, and involuntary assessment of physiological stress responses during community ambulation^27^. Yet, it is unknown if community ambulation can be more accurately predicted with objective, sensor-based measure of perceived challenge or through subjective balance confidence questionnaires. To successfully enhance community ambulation, it is essential to accurately identify individuals who perceive a high challenge of walking and assess how their perceived challenge may impact their daily walking activity in home and community environments.

Moreover, over the past two decades, the measurement of community ambulation has advanced from self-reported questionnaires to utilizing wearable sensors to measure daily steps^21,39,40^. However, as community ambulation is most widely defined as “independent mobility outside the home, which includes the ability to confidently negotiate uneven terrain, private venues, shopping centers and other public spaces”^21^, simply quantifying total daily steps does not accurately reflect this definition. To fully understand and correctly report post-stroke walking ability, it is vital to specifically quantify true community ambulation as only that which occurs outside the home. With improvement in Global Positioning System (GPS) technology, daily stepping activity can now be accurately parsed into home and community steps post-stroke^41–43^. Yet, it is unknown if perceived challenge of walking impacts community ambulation that specifically occurs outside-of-home. Analyzing the utility of wearable technology like accelerometers, GPS devices and skin conductance sensors may encourage the clinical use of consumer-friendly technological resources to quantify both perceived challenge of walking as well as home and community ambulation post-stroke.

Thus, the primary purpose of this study was to test differences in home and community ambulation between stroke survivors with lower and higher perceived challenge of walking. We hypothesized that compared to individuals with low perceived challenge of walking, those with high perceived challenge of walking would take fewer home and community steps, when perceived challenge of walking was measured *objectively* with SNS activation, but not when measured *subjectively* with the ABC scale.

## Methods

This cross-sectional study was conducted at the Malcolm Randall VA Medical Center, Gainesville, and Brooks Rehabilitation Clinical Research Center, Jacksonville, Florida between October 2019 and May 2021. Participants were: (1) at least six months post-stroke, (2) > 18 years of age, (3) community-dwelling, (4) able to follow 3-step command, (5) able to ambulate independently without physical assistance from another person, with or without an assistive or orthotic device, and (6) community ambulators. Participants were excluded for (1) a neurological diagnosis other than stroke, (2) history of intermittent claudication, (3) angina at rest or with minimal exertion, (4) history of COPD, (5) orthopedic conditions that limit mobility, and (6) ongoing physical rehabilitation services. Eligible participants signed a written informed consent form approved by the University of Florida Institutional Review Board. The procedures used in this study were approved by the University of Florida’s Institutional Review Board (IRB#201901748 and IRB#201900944) and adhere to the tenets of the Declaration of Helsinki. This study’s protocol is based on our previously published work^6,9,28,37^.

To characterize our participants clinical walking capacity, we assessed their gait speed and gait-related endurance. To obtain gait speed, participants walked on a GAITRite mat, an instrumented, computerized 4.98-m walkway, and completed two trials at their self-selected speed. Gait speed was calculated as the average of the two trials. The GAITRite has shown high concurrent validity in measuring hemiparetic gait speed when compared with 3-Dimentional motion capture system^44^. The Six-Minute Walk Test (6MWT) assessed participants’ gait endurance^45–47^, as they walked for six minutes around a 16-m walkway. The 6MWT has demonstrated excellent test–retest reliability (ICC: 0.95)^48^. Customary assistive and orthotic devices were permitted for assessments.

Perceived challenge of walking during daily home and community ambulation was assessed subjectively with the ABC Scale^24^. This 16-item self-report questionnaire assesses the stem question “How confident are you that you will not lose your balance or become unsteady when you…” on a scale of 0% (not confident at all) to 100% (completely confident). The ABC scale has high internal consistency (*Cronbach’s α* = *0.94*), excellent test–retest reliability (*ICC* = *0.85*) and has a moderately positive association with functional balance ability measured by the Berg Balance Scale (*ρ* = *0.36*) as well as gait speed *(ρ* = *0.48)* in individuals post-stroke^24^. The final score was calculated as a 16-item average, in which higher percentage values depicted higher balance confidence (lower perceived challenge).

SNS activity was measured from palmar sweat responses as participants walked a prescribed, laboratory-based indoor (lower challenge) and outdoor (higher challenge) course. All participants completed one trial of each task in the order outlined in Table 1. We chose not to randomize the order of the tasks as SNS responses may increase rapidly when performing a higher challenge task but may display a slower recovery to baseline if participants performed the higher challenge task first followed by the lower challenge task^49^. A waist belt-worn data acquisition unit (Flexcomp Infiniti, Thought Technology, Montreal, QC, Canada) recorded participants’ palmar sweat responses. Adhesive and disposable electrodes, with a conductive paste (0.5% saline in a neutral base) applied to the 10 mm Ag/AgCl recording surface, were securely placed on the proximal phalanges of both hands’ index and ring fingers^50^. An event-marking device connected to the data acquisition unit was manually activated by study personnel to insert markings in the data pertaining to key events of baseline resting, indoor, and outdoor walking tasks. Verbalization was limited to the provision of directions of the prescribed path, to reduce extraneous influence on SNS responses.

**Table 1:** Walking tasks sequence for indoor and outdoor environments.

Walking tasks	Distance	Instructions to participant
Indoor walking tasks		
Baseline resting	N/A	Sit in a relaxed and quiet position for 1 min
Typical walking	40 m	Walk in laps around a 15-m well-lit, unobstructed, level walkway
Typical walking	20 m	Walk in slightly crowded but levelled corridor, while making turns towards the exit of the building
Outdoor walking tasks		
Walking on sidewalk	50 m	Walk outdoors on a sidewalk towards ramp
Walking up two ramps	30 m	Walk up this ramp, turn and walk up the other ramp
Walking down two ramps	30 m	Make a U-turn and walk down these ramps
Walking on grass	10 m	Turn and walk on the grass
Walking on sidewalk	50 m	Walk on sidewalk towards the building entrance

*m* meters.

To accurately quantify true community ambulation as only that which occurs outside the home, participants wore an accelerometer (StepWatch Activity Monitor-4 (SAM)) along with a GPS device (GlobalSat DG-500) for seven days. The SAM demonstrates high test–retest reliability (ICC > 0.96)^51^ and high criterion validity (Pearson’s *r* = *0.96*) for post-stroke step counts in both indoor and outdoor environments^52^. The GlobalSat, a pager-sized GPS device^53–55^, recorded participants’ location at 5-s intervals on a micro SD card, viewed post-data collection with device-specific software^54^. GlobalSat GPS devices have been validated to study outdoor walking^53^ and have been previously used to quantify outdoor ambulation in people with stroke^41^. Participants wore the GPS device on a waist belt and the SAM on their non-paretic ankle during all waking hours except while bathing. Participants charged the GPS device nightly. To complement the GPS data during data cleaning, and analysis, participants completed a Trip Activity Log (TAL) (Supplemental Material#1, Fig. S1.1). One complete trip was operationally defined as leaving one’s home/yard space to go out in the community and returning home^5^. A trip may include visiting multiple types of locations such as going to a coffee shop, followed by grocery store and then a medical appointment before returning home. Participants were instructed to fill out the time they departed from home and arrived back home for each trip per day.

### Data analysis

Skin conductance signals were sampled at 32 Hz, and downloaded through Biograph Infiniti software (Thought Technology, Montreal, QC, Canada). Skin conductance was analyzed using MATLAB (v. R2019a; The Mathworks, Natick MA) with Ledalab v3.4.9. Raw data were down-sampled to 8 Hz followed by visual inspection for major signal artifacts that may be attributable to abrupt finger movements (e.g., forming a fist), tugging of wires or other unknown sources. Such major artifacts were indicated by rapid, high frequency fluctuations in their signal amplitude, inconsistent with the rate of amplitude change typically observed in electrodermal responses^6,9,28,37^. Relatively few outlying artifacts were identified and these were removed and replaced with linear interpolation (see example raw data plot in Supplementary Material#2, Fig. S2). Analyzed skin conductance signals were separated into skin conductance level (SCL) and skin conductance response (SCR) components using continuous decomposition analysis^28,50^. SCL indicates overall, slow-tonic responses to the challenges of walking whereas moments of acute unsteadiness and fear during walking are represented through the fast-phasic and high frequency changes in SCR^6,27^. An amplitude criterion of 0.05 microsiemens (μS) defined SCRs and minimized any movement artifacts^6^.

We determined change in SCL from simple indoor tasks to complex outdoor walking tasks as:

$$\Delta {\text{SCL}} = {\text{Walking}}\;{\text{maximum}}_{{{\text{Outdoor}}}} - {\text{Walking}}\;{\text{maximum}}_{{{\text{Indoor}}}}$$

Similarly, change in the rate of SCR from indoor to outdoor walking tasks was calculated as:

$$\Delta {\text{SCR}} = {\text{Rate}}\;{\text{of}}\;{\text{SCR}}\;{\text{during}}\;{\text{outdoor}}\;{\text{walking}} - {\text{Rate}}\;{\text{of}}\;{\text{SCR}}\;{\text{during}}\;{\text{indoor}}\;{\text{walking}}$$

The rate of SCRs was defined as the number of SCRs detected during a recording period, divided by the duration of the recording period^6^. We used the mean of each of the three perceived challenge of walking variables (∆SCL, ∆SCR and ABC) to categorize participants into higher and lower perceived challenge groups as accurate cut-offs for these variables have not been established in the literature. By evaluating the accuracy of each of the three variables, the results of this categorization would help clinicians and rehabilitation scientists choose the most accurate variable when predicting community ambulation post-stroke.

For quantification of community ambulation, the SAM provided total strides/day over 10-s intervals in a Microsoft Excel format^56^. To obtain total steps/day, the number of strides were doubled, and then summed. To ensure compliance, step data was plotted and visually inspected for at least 8 h of wear time per day (see example plots in Supplemental Material#1, Fig. S1.2)^57^. Any day that did not meet this criterion was excluded from the analysis. Location data (latitude, longitude, timestamp) from the GPS device was downloaded via GlobalSat software in a Microsoft Excel format. Using time stamp synchronization in MATLAB through a custom code, we mapped the SAM-derived stepping activity onto the location data from the GPS device. The latitude and longitude for the patient’s residential address was derived from Google Maps. Home was operationally defined as an individual’s geographical residential address with an 85-m surrounding perimeter. The 85-m radius accounted for average yard space^58^ and any erroneous spikes (i.e., noise) in the GPS signals when indoors. Each participant’s walking location was categorized as home or community (location away from home). The GPS file was visually inspected for any missing data that exceeded 30 min. Any missing GPS data was compared with the participant’s TAL and characterized as ‘missing at home’, ‘missing in community’, or ‘missing while in transition between home and community’. For missing GPS data while the participant was at home, MATLAB automatically characterized steps during this period as ‘home steps’. If the missing GPS data coincided completely with the participant’s time outside of home, MATLAB interpolated the data between the previous and next locations in the community, classifying all steps taken during this missing period as ‘community steps’. If GPS data was missing while the participant was in transition between home and community, and if steps taken during this period were less than 10% of total steps for the day, MATLAB interpolated the step data between the previous and next GPS location. However, if steps taken during the ‘missing while in transition’ period exceeded 10% of the total steps for that day, the entire day was excluded from the analysis to ensure more than 90% accuracy in classifying steps as home and community (see SAM-GPS data analysis algorithm in Supplemental Material#1, Fig. S1.3). Data included in the analysis were obtained from devices worn for at least four days and eight hours per day. Home and community steps/day were extracted and averaged for the recording period. All authors have full access to study data and take responsibility for its integrity.

### Statistical analysis

We used the Kolmogorov–Smirnov and Shapiro–Wilk tests^59^ to determine the normality of all variables. To examine any differences in clinical or demographic characteristics with the ΔSCL, ΔSCR and ABC groups, we conducted independent t-tests. To assess the relationship between ΔSCL, ΔSCR, ambient temperature and relevant demographic characteristics, we conducted Pearson product-moment correlation and Spearman’s Rho correlation tests for the continuous and categorical variables, respectively. To examine differences between indoor and outdoor walking in SCR and SCL within each participant, we conducted repeated measures ANOVA tests with ambient temperature as a covariate. To assess for differences in home and community ambulation between the groups of higher and lower perceived challenge, we conducted three separate MANOVA models (one model for each classification variable: ∆SCL, ∆SCR and ABC). Any significant main effects were interpreted with follow-up univariate analyses with Bonferroni corrections. We used the Box’s M test (Box’s Test for Equivalence of Covariance Matrices) to test if the covariation between dependent variables was equal across groups. After confirming multivariate normality assumptions (a non-significant Box’s M) and non-redundancy of independent variables, we proceeded with statistical analysis, using the *Statistical Package for the Social Sciences* (*SPSS, Version 24*) with the significance level set at alpha equal to 0.05. Power analysis suggested a total of 29 subjects to achieve 80% power (Details in Supplementary Material#3). To account for non-compliance and technical issues, we enrolled a total of 40 individuals in this study. Any differences in demographic and clinical characteristics between the included and excluded participants were tested through independent t-tests (continous variables) and Chi-square tests (categorical variables).

## Results

Of the forty individuals enrolled, eleven were excluded from the final analysis due to: non-compliance with wearing the SAM/GPS devices (n = 2), insufficient GPS data (n = 2), technical difficulties with recording skin conductance (n = 2) and poor-quality of processed SNS signals with extremely low amplitude (n = 5). Thus, the final analysis included 29 participants. Demographic and clinical characteristics did not differ between the included and excluded participants (Table 2).

**Table 2:** Demographic and clinical characteristics.

Characteristic (n = 29)	Included participants (n = 29) (Mean (SD) or frequency)	Excluded participants (n = 11) (Mean (SD) or frequency)	P values for differences between included and excluded participants
Mean age (yrs)	61.00 (8.79)	66.6 (10.8)	*p* = *0.14*
Gender	12 Females/ 17 Males	5 Females/ 6 Males	*p* = *0.81*
Mean time post-stroke (yrs)	5.01 (3.31)	3.4 (2.94)	*p* = *0.19*
Side of Paresis (R/L)	12 Right/ 17 Left	5 Right/ 6 Left	*p* = *0.81*
Race	White: (20/29) 69%; Black: (9/29): 31%	White: (4/11) 36%; Black: (7/11) 64%	*p* = *0.06*
Uses Assistive Device^Ŧ^	Yes: (20/29) 69%	Yes (8/11) 73%	*p* = *0.81*
Tested during COVID-19 (July 2020 to April 2021)	Yes: 48.3%	Yes: 73%	*p* = *0.17*
Gait Speed (m/s)	0.69 (0.32)	0.69 (0.32)	*p* = *1.000*
Six-Minute Walk Test (m)	243.81 (100.85)	267.76 (73.86)	*p* = *0.48*

^Ŧ^Types of assistive devices: Single point cane, Quad cane, Hemi-walker, rolling walker *m* meters, *m/s* meters per second, *yrs* years.

**Figure 1:**
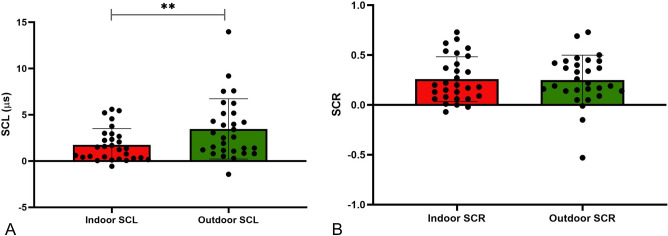
Difference between indoor and outdoor sympathetic nervous system activity measurements using repeated measures ANOVA tests with ambient temperature as a covariate. (**A**) Outdoor SCL (green) is significantly higher than indoor SCL (red), after accounting for ambient temperature (**p < 0.001) [SCL_(indoor or outdoor)_ = Walking maximum − Resting minimum]. (**B**) Outdoor SCR (green) is not significantly different from indoor SCR (red) [SCR_(indoor or outdoor)_ = Rate of SCR during walking − Rate of SCR during rest].

Most participants (69.4%) used an assistive device in their non-paretic hand for balance support during walking trials (Table 2). Since pressure and contact with the assistive device can significantly alter skin conductance^50^, we analyzed SNS signals from the paretic hand only. The ambient outdoor temperature for all participants ranged between – 2 °C to 34 °C (average 21° ± 8 °C) and had a moderately positive association with ΔSCL (*r* = *0.48, p* = *0.008*), but not with ΔSCR (see Supplemental Material#3, Table S3). The interaction between ambient outdoor temperature (covariate) and indoor and outdoor SCL values within each participant was significant (Wilks’ Λ = 0.77; F (27, 1) = 8.21; *p* = *0.008*, Effect size *ŋ2* = *0.233*). Even after accounting for ambient temperature, outdoor SCL (3.47 ± 3.25 µs) was significantly higher than indoor SCL (1.75 ± 1.77 µs) (*p* < *0.001*), suggesting a higher perceived challenge in the outdoor walking environment, as compared to indoor (Fig. 1A). In contrast, outdoor SCR (0.25 ± 0.25) did not differ from indoor SCR (0.26 ± 0.23) (*p* = *0.83*) in our cohort (Fig. 1B).

The categorization of higher and lower perceived challenge groups based on mean ∆SCL, ∆SCR and ABC values is depicted in Table 3. Albeit non-significant, all lower perceived challenge groups (lower ΔSCL, lower ΔSCR, higher ABC) showed trends of faster gait speed and greater distance covered in the 6MWT, as compared to all higher perceived challenge groups (higher ΔSCL, higher ΔSCR, lower ABC groups) with small to medium effect sizes as depicted by *Hedges’ g* in Table 4.

**Table 3:** Group division based on the average values of the three perceived challenge of walking variables.

Perceived walking challenge variable	Higher perceived challenge of walking group	Lower perceived challenge of walking group
ΔSCL (n = 29)	ΔSCL ≥ 1.7 µS (n = 12)	ΔSCL < 1.7 µS (n = 17)
ΔSCR (n = 29)	ΔSCR ≥ 0.2 (n = 16)	ΔSCR < 0.2 (n = 13)
ABC (n = 29)	ABC < 72% (n = 13)	ABC ≥ 72% (n = 16)

*ΔSCL* skin conductance level (outdoor–indoor), *μS* microSiemens, *ΔSCR* skin conductance—response (outdoor–indoor), *ABC* activities-specific balance confidence scale.

**Table 4:** Difference in demographic characteristics and clinical outcomes (Mean (SD)) in the groups of higher and lower perceived walking challenge.

Variables	High ΔSCL Group (n = 12)	Low ΔSCL group (n = 17)	High ΔSCR group (n = 16)	Low ΔSCR group (n = 13)	Low ABC group (n = 13)	High ABC group (n = 16)
Age (yrs)	60.8 (8.5)	61.1 (9.2)	60.7 (7.8)	61.4 (10.1)	61.4 (7.9)	60.7 (9.7)
Time since stroke (yrs)	3.7 (1.8)	5.9 (3.8)	4.5 (2.7)	5.6 (3.9)	5.0 (3.0)	5.0 (3.6)
ABC (%)	73.1 (14.1)	70.2 (21.6)	71.6 (14.1)	71.3 (23.6)	54.6 (13.2)	85.1 (7.8)**
Home Steps/Day	1859 (1392)*	3098 (1655)	2187 (1505)	3075 (1737)	2519(1597)	2638 (1735)
Community steps/day	1256 (987)*	2132 (1154)	1453 (1114)	2160 (1127)	1685 (1134)	1839 (1206)
Gait Speed (m/s)	0.60 (0.28)	0.74 (0.34)	0.63 (0.29)	0.75 (0.36)	0.62 (0.30)	0.73 (0.34)
*Hedges’ g*	*0.39*		*0.37*		*0.34*	
6MWT (m)	227.3 (86.1)	255.4 (111.1)	226.1 (88.2)	265.5 (114.3)	212.3 (95.4)	269.3 (100.7)
*Hedges’ g*	*0.28*		*0.39*		*0.58*	

*ΔSCL* skin conductance level (outdoor–indoor), *ΔSCR* skin conductance response (outdoor–indoor), *ABC* activities-specific balance confidence scale, *6MWT* six-minute walk test, *m* meters, *m/s* meters per second, *yrs* years.

**p < 0.001 as per independent t-test between higher and lower ABC group.

*p < 0.05, high ΔSCL group significantly different from low ΔSCL group.

**Figure 2:**
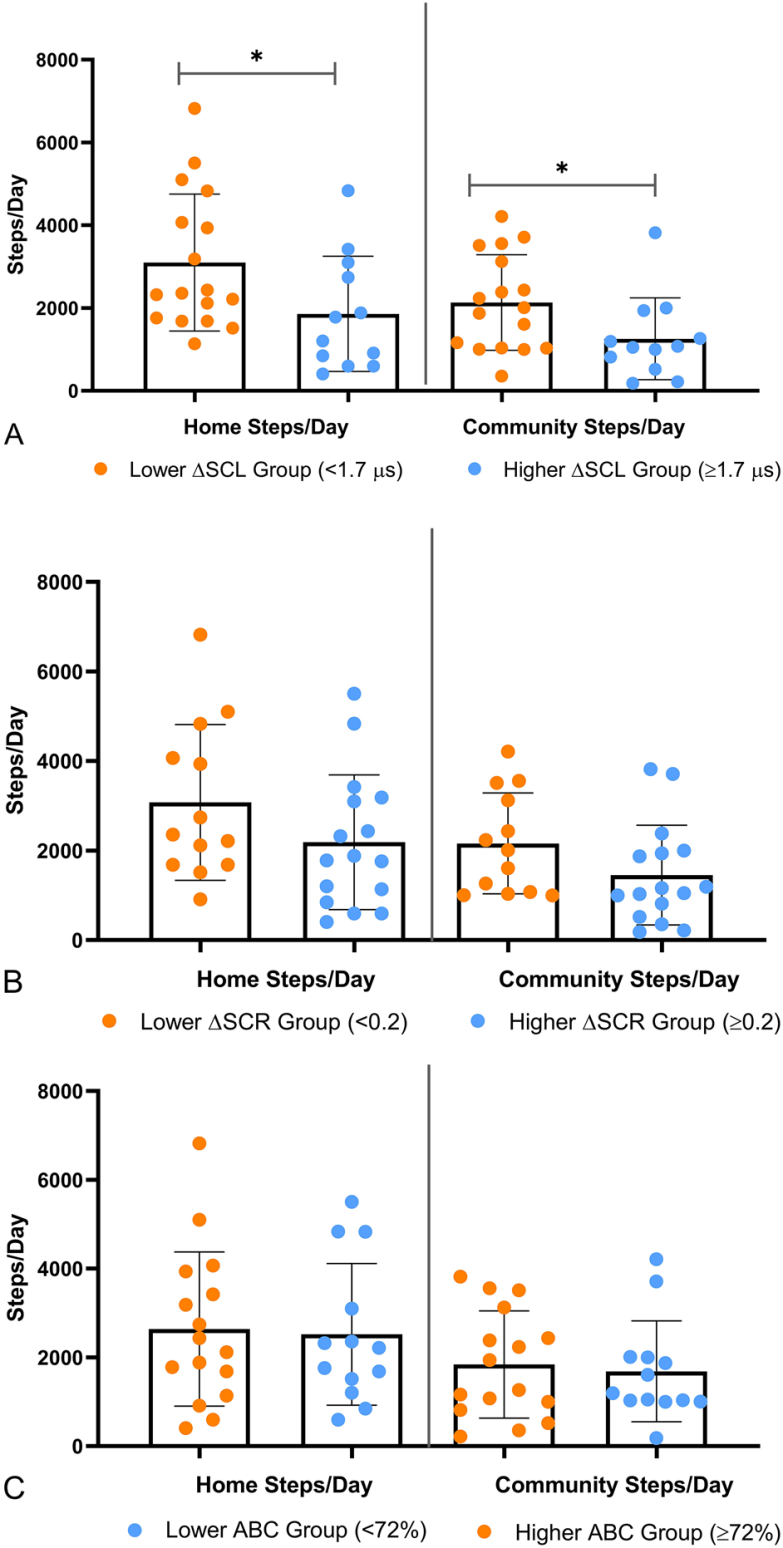
Difference in home steps and community steps/day between higher and lower perceived challenge of walking groups. (**A**) Higher ΔSCL group (blue) took significantly fewer home steps/day (*Hedges’ g* = *0.80*) and community steps/day *(Hedges’ g* = *0.80)* than the lower ΔSCL group (orange) (*p < 0.05)*.* (**B**) The higher ΔSCR group (blue) showed trends towards reduced home steps/day (*Hedges’ g* = *0.55*) and community steps/day (*Hedges’ g* = *0.63*) than the lower ΔSCR group (orange). (**C**) Higher ABC group (orange) did not differ in home steps/day (*Hedges’ g* = 0.07) and community steps/day (*Hedges’ g* = 0.13) from the lower ABC group (blue). ABC-Activities-specific Balance Confidence Scale.

Home and community steps/day differed significantly between the higher and lower ΔSCL groups (Main effect: Wilks’ Λ = 0.752; F(26, 2) = 4.29; *p* = *0.024*, Effect size *ŋ*^*2*^ = *0.248*). Follow-up univariate analysis showed that higher ΔSCL group took significantly fewer daily steps at home and in the community than the lower ΔSCL group, (Table 5, Fig. 2A). In contrast to ΔSCL groups, home and community steps/day did not differ significantly between the higher and lower ΔSCR groups (Main effect: Wilks’ Λ = 0.852; F(26, 2) = 2.25; *p* = *0.12*, Effect size *ŋ*^*2*^ = *0.148*). Although non-significant, as compared to lower ΔSCR group, the higher ΔSCR group showed trends towards reduced home and community steps/day with medium effect sizes (Table 5, Fig. 2B). Home and community steps/day did not differ significantly between the higher and lower ABC groups (Main effect: Wilks’ Λ = 0.99; F(26, 2) = 0.07; *p* = *0.93*, Effect size *ŋ*^*2*^ = *0.005)* (Table 5, Fig. 2C).

**Table 5:** Difference between higher and lower perceived challenge of walking groups in home steps/day and community steps/day.

	Higher versus lower ΔSCL groups	Higher versus lower ΔSCR groups	Lower versus higher ABC groups
Home steps/day			
Mean difference in home steps/day (SE)	1239 (585)*	888 (602)	119 (625)
95% CI	38–2440	-347 to 2123	-1165 to 1403
Effect size	0.80	0.55	0.07
Community steps/day			
Mean difference in community steps/day (SE)	876 (410)*	707 (418)	153 (439)
95% CI	33–1719	− 150 to 1565	− 746 to 1054
Effect size	0.80	0.63	0.13

*ΔSCL* skin conductance level (outdoor–indoor), *ΔSCR* skin conductance response (outdoor–indoor), *ABC* activities-specific balance confidence scale. *p < 0.05; Significantly different from lower ΔSCL group.

## Discussion

This study aimed to test the hypothesis that individuals with high perceived challenge of walking would take fewer home and community steps than those with high perceived challenge of walking, when perceived challenge of walking was measured *objectively* with SNS activation, but not when measured *subjectively* with the ABC scale. Consistent with the hypothesis, individuals post-stroke who demonstrated higher perceived challenge of walking took fewer daily home and community steps than those with lower perceived challenge of walking, when perceived challenge of walking was quantified *objectively*, but not *subjectively*.

Our study provides unique evidence regarding the utility of assessing SNS responses during challenging walking tasks in differentiating between GPS-based daily home and community ambulation levels post-stroke. Measuring SNS responses to challenging walking tasks through wearable sensors provided an objective and unbiased assessment of how stroke survivors in our cohort perceived complex walking tasks as well as how this perception impacted their daily home and community steps. In the present study, individuals with above average increase in SCL between indoor and outdoor environments took significantly fewer home and community steps per day than individuals with below average increase in SCL. A similar trend of reduced home and community ambulation was also observed in the higher ΔSCR group, as compared to the lower ΔSCR group, with medium effect sizes (Table 5, Fig. 2A,B).

Previous reports have concluded that elevated autonomic stress responses are associated with decreased physical activity and increased sedentary time in healthy men and women^60–62^. This is the first study to suggest that SNS responses impact walking activity at home for individuals post-stroke. Experiencing a higher perceived challenge of walking may have negatively impacted our participants’ ability, intention and confidence to walk around at home^38^. On the other hand, in the presence of lower perceived challenge of walking one may be more apt to walk in the home to do daily chores, as well as complete tasks in one’s yard, such as going to the mailbox, or taking out trash, thus contributing to increased daily home steps per day^63^.

Similar to home ambulation, higher perceived challenge of walking, as evaluated by physiological measures, also hindered community ambulation in our participants. Previous research has reported increased SNS responses in older adults and in individuals post-stroke as they walked in complex environments^6,9,37^. Additionally, stroke survivors who demonstrated increased SNS responses during challenging walking tasks also exhibited cautious and slow gait patterns along with poorer task performance in previous reports^6,9,37^. Indeed, in our study, individuals with higher perceived challenge of walking demonstrated trends of slower gait speeds and reduced 6MWT distances as compared to those in the lower perceived challenge of walking. Since community ambulation requires one to walk for longer distances^64^ with confidence and adaptability to negotiate varied environments such as sidewalks with unanticipated crowds and uneven terrains like grass, and ramps^5,21,65^, participants within the high ΔSCL group may have found it difficult to walk in these complex scenarios, leading to disengagement or avoidance of community ambulation^8^. Furthermore, in a recent study, stroke survivors reported increased perceived level of walking difficulty during challenging walking tasks, similar to those conducted in the present study, like walking on grass and ramps, as compared to walking on a level surface^7^. Our findings support previous qualitative studies in which stroke survivors voiced their anxiety, insecurity and fear of falling as barriers to walking outdoors and gait-related participation^38,66^. With the advantage of wearable sensors, our results suggest that such heightened emotional and physiological stresses of challenging walking tasks may be more accurately measured through skin conductance than self-reported balance confidence and could differentiate between daily community ambulation levels in individuals with stroke.

In contrast, participants with higher and lower ABC scores (lower and higher perceived challenge of walking, respectively) did not differ in their daily home and community steps. It is plausible that our participants’ self-reported ABC scores may not have aligned with their actual confidence and ability to ambulate in their home and community environments, and may have been impacted by individual personality traits, culture, introspective abilities and literacy levels^26^. Individuals in the higher and lower ΔSCL and ΔSCR groups reported strikingly similar average ABC scores, despite demonstrating markedly different SNS responses while performing some of the ABC scale items such walking up and a down a ramp, uneven terrain, and outdoor sidewalks (Table 4). Like the ΔSCL and ΔSCR groups, the low ABC group demonstrated trends of slower gait speeds and reduced 6MWT distances than the high ABC group. However, unlike the SNS groups, these differences in clinical walking capacity did not translate to home and community environments for the subjective ABC groups, further highlighting the unique impact of SNS responses on community ambulation in our cohort. Moreover, only half of the items on the ABC scale are specifically related to walking outside one’s home in the community and some of these were not applicable to our cohort living in Florida, such as walking on icy sidewalks^24^. These characteristics of the ABC scale may have led to discrepancies between the subjective and objective measurement of perceived challenge of walking and impacted the ABC scale’s utility to distinguish between true community ambulation levels post-stroke. Similar discrepancies between self-reported ABC scores and SNS responses to challenging walking tasks have been stated previously in individuals with stroke^6^ and lower limb amputation^67^. Our findings are in conflict with previous reports that suggested a positive association between self-reported balance confidence and community ambulation^14^, community reintegration^18,19,68^ and daily walking activity post-stroke^15,17,69,16^. However, these previous studies examined community ambulation either only through self-report questionnaires^14,18,19,68^ or through total daily steps, not parsed into location of walking activity^13,15,17^. Like self-reported balance confidence, self-reported community ambulation may be prone to subjective biases and recall errors. Moreover, as community ambulation is defined as “independent mobility outside the home”^21^, simply quantifying total daily steps does not accurately reflect this definition. Thus, the inclusion of accelerometer and GPS technology strengthened the present study, compared to previous studies, to accurately examine community ambulation levels without subjective biases and uniquely parsed total daily steps specifically into home and community steps/day. Our results suggest that rehabilitation professionals should exercise caution when predicting community ambulation status merely based on self-reported balance confidence in patients post-stroke. This study provides vital insights to clinicians and rehabilitation professionals on the importance of assessing both community ambulation and perceived challenge of walking using objective, real-time measures.

### Limitations

We studied participants’ SNS activation as they traversed an outdoor walking path designed to mimic a typical community environment, yet provided close supervision to assure participants’ safety. However, stress responses may differ in a true real-world, community-based environment such as a mall or park where stroke survivors may walk independently, without supervision. Due to limited sensitivity of skin conductance measurement in accurately distinguishing various walking subtasks such as walking on grass or ramp^9,37^, we analyzed our outdoor walking task as one entity. It is possible that some subtasks may have had a larger contribution to outdoor SCL and SCR than others. Additionally, our findings may have been influenced by factors other than complexity of walking tasks as we did not randomize our task order. However, as described before, performing lower challenge tasks before higher challenge tasks was necessary to accurately detect any increase in SNS responses^49^. Moreover, as true cut-offs for categorizing individuals into higher and lower perceived challenge groups have not been established in the literature, we used the mean of each of the three variables to compare subgroups. Further research is warranted to establish accurate cut-off scores to classify individuals with stroke into higher or lower perceived challenge groups. Furthermore, as this study was conducted in the subtropical climate of Florida, our results may not generalize to geographic areas with extremely cold climates. Lastly, at the time of this study, the ABC scale was the only validated questionnaire that assesses balance confidence during both home and community ambulation post-stroke. Further investigation of other validated questionnaires of perceived challenge of walking and their association with community ambulation is warranted.

## Conclusion

In the present study, stroke survivors with higher perceived challenge of walking in complex outdoor walking activities, as objectively measured by skin conductance, took fewer daily steps in both home and community, as compared to those with lower perceived challenge of walking. The impact of perceived challenge of walking on daily home and community ambulation was more accurately evaluated through SNS responses than a self-reported questionnaire in our cohort. As stroke survivors may over- or under-estimate their balance confidence in daily life, rehabilitation specialists should be aware of the potential limitations of predicting home and community ambulation levels solely based on subjective assessments. With increased advances in consumer-friendly wearable technology^70,71^, researchers should consider developing and validating clinically applicable, consumer-friendly, wearable devices to examine real-time SNS responses during challenging walking tasks as well as to quantify daily home and community ambulatory activity. While our previous work demonstrated reduced SNS activity following gait rehabilitation^37,72^, future research may investigate the impact of diminished perceived challenge of walking on daily home and community ambulation.

### Supplementary Information

Supplementary Information 1.

Supplementary Information 2.

Supplementary Information 3.

## Data Availability

The data that support the findings of this study are available on request from the corresponding author, [KB]. The data are not publicly available due to their containing information that could compromise the privacy of research participants (participant’s home addresses from GPS devices).

## Abbreviations

ABC: Activities-specific balance confidence
SNS: Sympathetic nervous system
SCL: Skin conductance levels
SCR: Skin conductance responses
6MWT: Six-minute walk test
SAM: Step activity monitor
TAL: Trip activity log
