# Supplementary material for: Sympathetic nervous system responses during complex walking tasks and community ambulation post-stroke
Source: Sci Rep. 2023 Nov 16;13:20068. doi: 10.1038/s41598-023-47365-5 (PMC10654447; doi:10.1038/s41598-023-47365-5)
Supplement: Supplementary file 1 — Supplementary Information 1. [file 41598_2023_47365_MOESM1_ESM.pdf]

Supplemental Material – 1

Sympathetic Nervous System Responses during Complex Walking Tasks and Community Ambulation Post-stroke

Kanika Bansal, PT, MPT, PhD[1]*; David J. Clark, ScD[2,3]; Emily J. Fox, DPT, MHS, PhD[2,4]; and Dorian K. Rose, PT, PhD[2,3,4]

[1]University of Mount Union, Alliance, OH, USA; [2]University of Florida, Gainesville, FL, USA; [3]Brain Rehabilitation Research Center, Malcolm Randall Veterans Affair Medical Center Gainesville, FL, USA; [4]Brooks Rehabilitation, Jacksonville, FL, USA.

Figure S1.1: Trip Activity Log (X 30 pages):

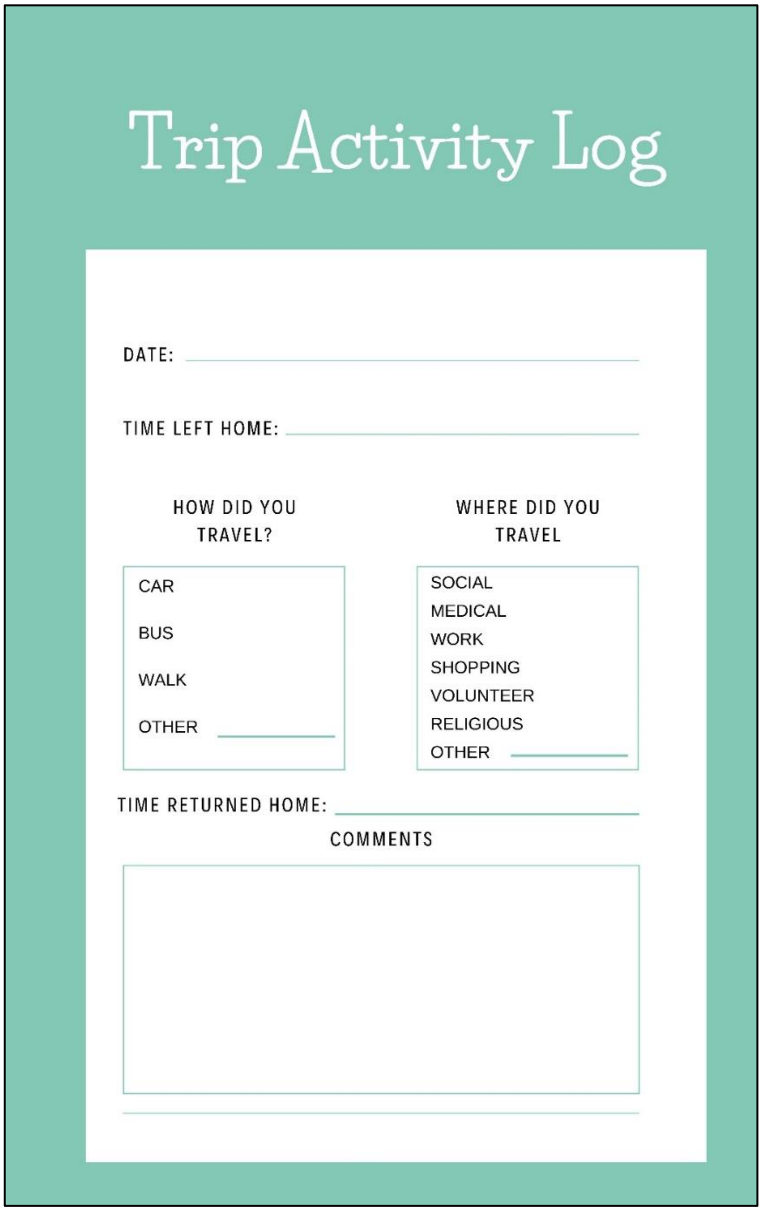
Trip Activity Log

DATE: ______

TIME LEFT HOME: ______

HOW DID YOU TRAVEL?

CAR
BUS
WALK
OTHER ______

WHERE DID YOU TRAVEL

SOCIAL
MEDICAL
WORK
SHOPPING
VOLUNTEER
RELIGIOUS
OTHER ______

TIME RETURNED HOME: ______

COMMENTS

Figure S1.2: Example step data plots to confirm whether participant wore the Step Activity Monitor (SAM) for more than 8 hours/day.

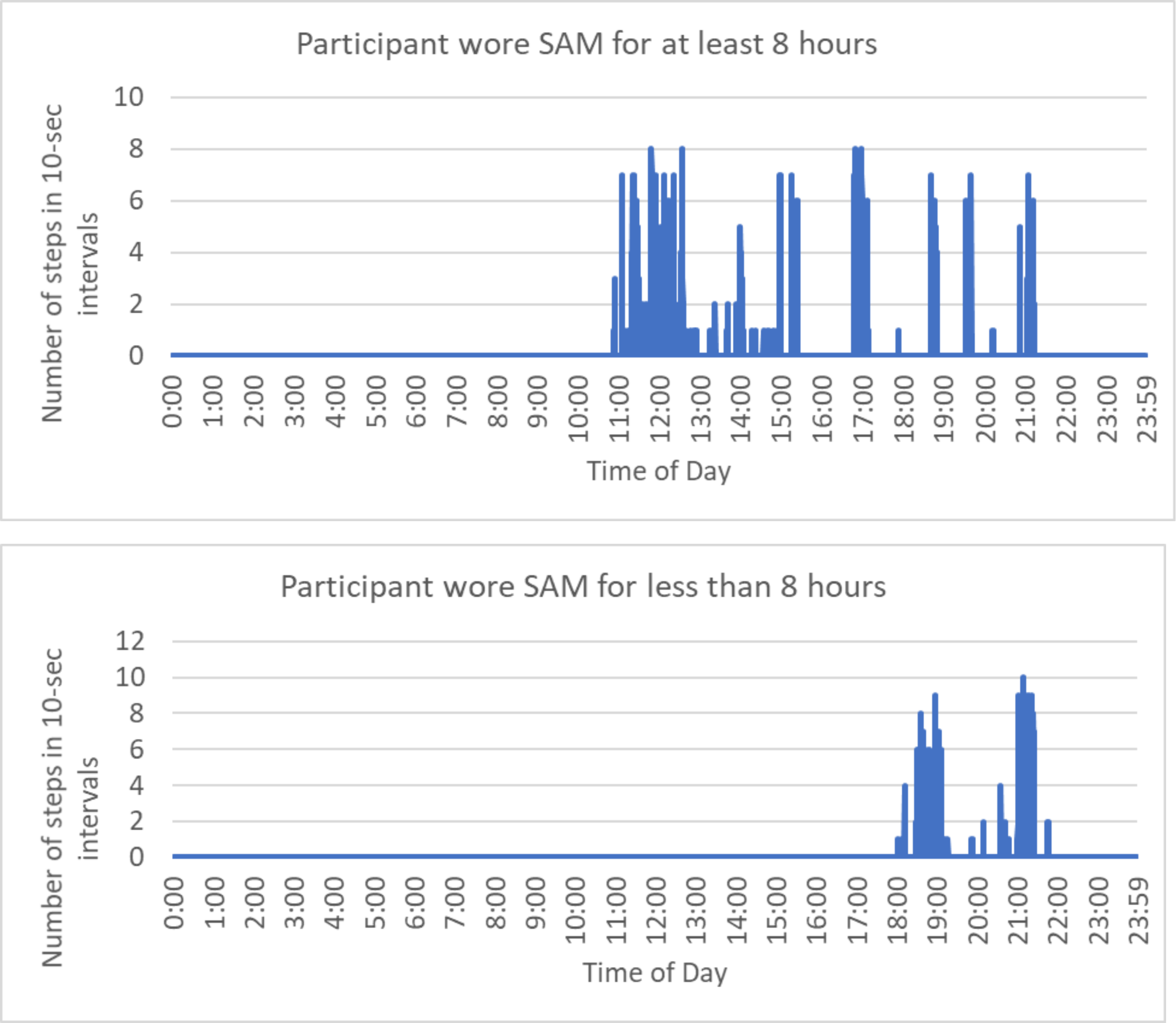

Figure S1.3: Algorithm for SAM-GPS missing data processing

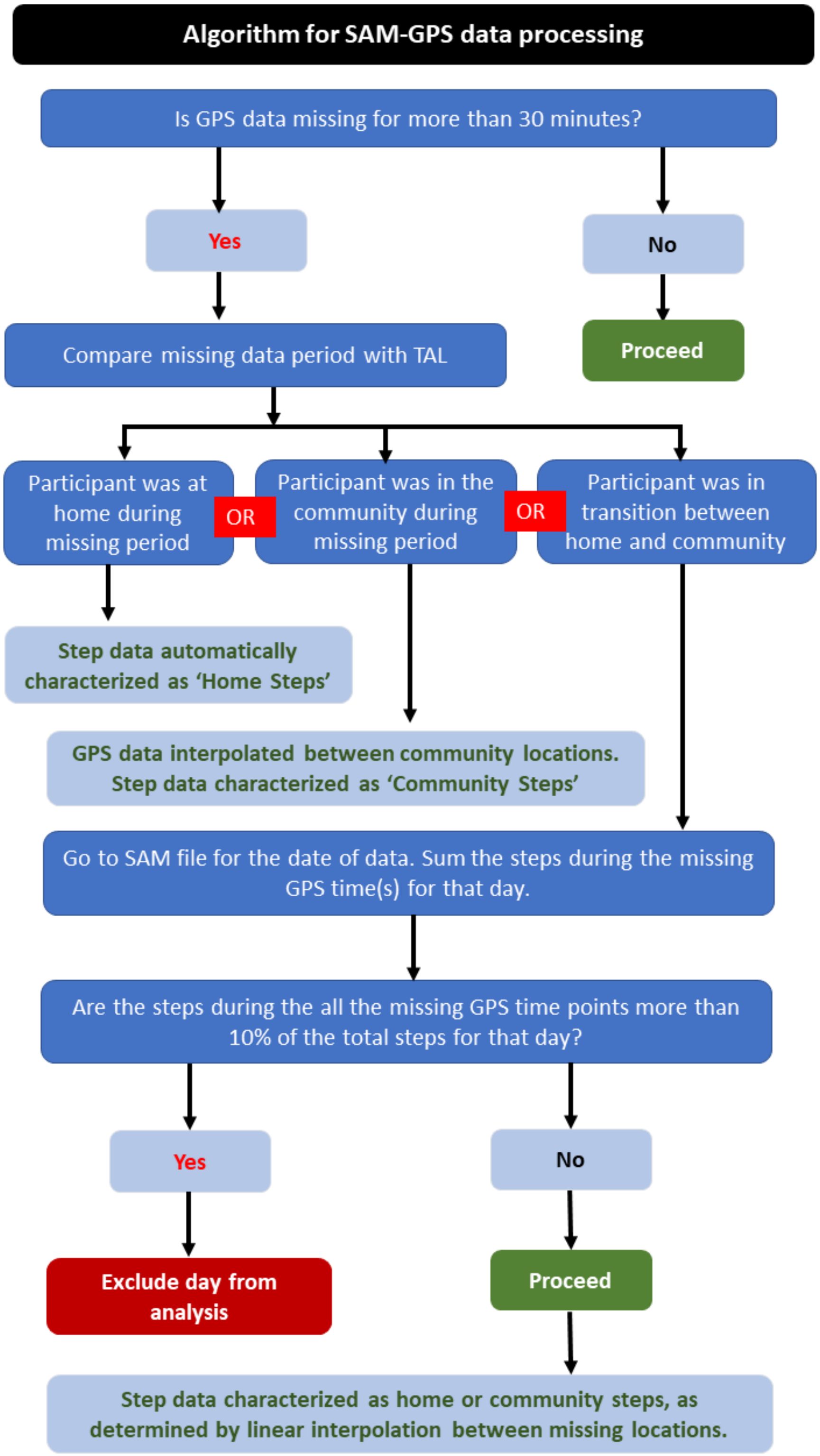

SAM: Step Activity Monitor, GPS: Global Positioning System, TAL: Trip Activity Log
