# Supplementary material for: Sympathetic nervous system responses during complex walking tasks and community ambulation post-stroke
Source: Sci Rep. 2023 Nov 16;13:20068. doi: 10.1038/s41598-023-47365-5 (PMC10654447; doi:10.1038/s41598-023-47365-5)

Supplemental Material – 2

Sympathetic Nervous System Responses during Complex Walking Tasks and Community Ambulation Post-stroke

Kanika Bansal, PT, MPT, PhD[1]*; David J. Clark, ScD[2,3]; Emily J. Fox, DPT, MHS, PhD[2,4]; and Dorian K. Rose, PT, PhD[2,3,4]

[1]University of Mount Union, Alliance, OH, USA; [2]University of Florida, Gainesville, FL, USA; [3]Brain Rehabilitation Research Center, Malcolm Randall Veterans Affair Medical Center Gainesville, FL, USA; [4]Brooks Rehabilitation, Jacksonville, FL, USA.

Figure S2: Example raw data plot to visually identify signal artifacts.

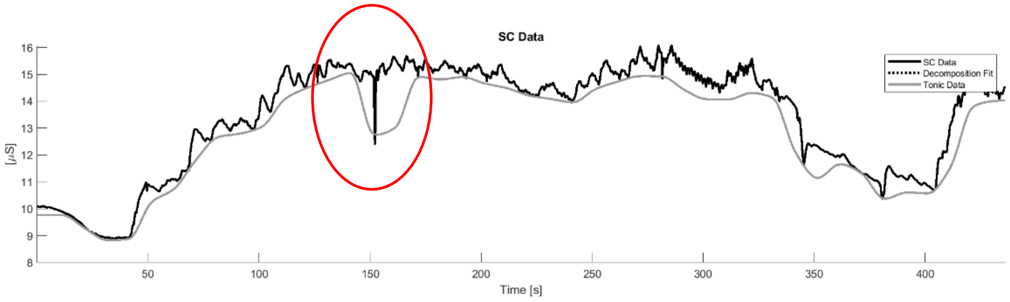

Supplement: Supplementary file 2 — Supplementary Information 2. [file 41598_2023_47365_MOESM2_ESM.pdf]
