# Supplementary material for: Sympathetic nervous system responses during complex walking tasks and community ambulation post-stroke
Source: Sci Rep. 2023 Nov 16;13:20068. doi: 10.1038/s41598-023-47365-5 (PMC10654447; doi:10.1038/s41598-023-47365-5)
Supplement: Supplementary file 3 — Supplementary Information 3. [file 41598_2023_47365_MOESM3_ESM.pdf]

Supplemental Material – 3

Sympathetic Nervous System Responses during Complex Walking Tasks and Community Ambulation Post-stroke

Kanika Bansal, PT, MPT, PhD[1*]; David J. Clark, ScD[2,3]; Emily J. Fox, DPT, MHS, PhD[2,4]; and Dorian K. Rose, PT, PhD[2,3,4]

[1]University of Mount Union, Alliance, OH, USA; [2]University of Florida, Gainesville, FL, USA; [3]Brain Rehabilitation Research Center, Malcolm Randall Veterans Affair Medical Center Gainesville, FL, USA; [4]Brooks Rehabilitation, Jacksonville, FL, USA.

Power Analysis: Power analysis for this study is based on our pilot study with 12 participants. We conducted a MANOVA model to analyze the difference between home steps and community steps in the higher and lower ΔSCL groups. The main effect between the groups was not significant (Pillai's trace=0.268, *p=0.18*) in the pilot study. Based on this data, we needed a total of 29 subjects to achieve 80% power while controlling the type I error to 0.05 level, as calculated in G*Power software (Düsseldorf, Germany). To account for non-compliance and technical issues, we planned to recruit a total of 40 individuals in this study.

Table S3: Bivariate relationship between ΔSCL, ΔSCR, ambient temperature and demographic characteristics.

Variable	ΔSCL (r)	ΔSCR (r)
Temperature	0.48*	-0.05
Age	-0.15	-0.08
Side of Paresis (Left=1, Right=2)	0.06	0.22
Gender (Female=1, Male=2)	0.07	-0.25
ΔSCR	0.56**	___________

SCR- Skin Conductance Response; SCL- Skin Conductance Level. *p<0.05; **p=0.001; Pearson's Moment and Spearman's Rho tests for continuous and categorical variables, respectively.
